# Living with palindromic rheumatism: a qualitative interview study

**DOI:** 10.1093/rap/rkaf138

**Published:** 2025-12-02

**Authors:** Lara S Chapman, Zahira P Latif, Rebecca J Stack, Anne-Maree Keenan, Hanna Gul, Paul Emery, Kulveer Mankia, Karim Raza, Heidi J Siddle

**Affiliations:** Leeds Institute of Rheumatic and Musculoskeletal Medicine, University of Leeds, Leeds, UK; UCL Consultants Ltd, University College London, London, UK; Three Counties Medical School, University of Worcester, Worcester, UK; School of Healthcare, University of Leeds, Leeds, UK; NIHR Leeds Biomedical Research Centre, Leeds Teaching Hospitals NHS Trust, Leeds, UK; Leeds Institute of Rheumatic and Musculoskeletal Medicine, University of Leeds, Leeds, UK; Leeds Institute of Rheumatic and Musculoskeletal Medicine, University of Leeds, Leeds, UK; NIHR Leeds Biomedical Research Centre, Leeds Teaching Hospitals NHS Trust, Leeds, UK; Leeds Institute of Rheumatic and Musculoskeletal Medicine, University of Leeds, Leeds, UK; NIHR Leeds Biomedical Research Centre, Leeds Teaching Hospitals NHS Trust, Leeds, UK; Department of Rheumatology, Institute of Inflammation and Ageing, University of Birmingham, Birmingham, UK; Leeds Institute of Rheumatic and Musculoskeletal Medicine, University of Leeds, Leeds, UK

**Keywords:** palindromic rheumatism, patient perspectives, at-risk, RA, qualitative, interviews, prevention

## Abstract

**Objectives:**

Palindromic rheumatism (PR) is an unpredictable and under-researched inflammatory condition, and patients with PR are at risk of developing inflammatory arthritis (IA). This study aimed to explore patients’ perceptions and experiences of living with PR, including symptoms, impact, treatment outcomes and potential progression to IA.

**Methods:**

Patients were recruited from ongoing cohort studies identifying individuals at risk of developing IA. Semi-structured interviews were conducted at two UK sites. Data were analysed using reflexive thematic analysis. Patient research partners co-produced the interview schedule and contributed to coding decisions.

**Results:**

Eight patients were interviewed. Three themes (seven subthemes) were identified: experiencing symptoms (symptoms, perceptions of triggers, referral experiences); impact of symptoms (activity limitations, psychological impact); treatment expectations and knowledge seeking (treatment outcomes and progression, information and support needs). Symptom severity was likened to that associated with severe physical injury, and PR impacted on daily activities and caused psychological distress, but referral delays were frequently reported. Patients expressed concerns about taking medication for PR, primarily due to side effects. Most highlighted a lack of information about PR (e.g. medication options and self-management advice) but varied in how much they wanted to understand about PR progression and treatment options.

**Conclusion:**

This study captured valuable insights into the perceptions and experiences of PR, from the perspective of patients. Findings highlight the severity of symptoms and impact of the condition. Further work to standardize classification criteria and outcome measurement in PR is critical to facilitate meaningful clinical trials in this area.

Key messages

- Palindromic rheumatism patients report severe and unpredictable symptoms that cause significant activity limitations and psychological impact.
- Diagnostic delays are common in palindromic rheumatism; patients need earlier referral to rheumatology specialist services.
- Findings can inform the design of meaningful clinical trials in this area.

## Introduction

Palindromic rheumatism (PR) is an inflammatory condition characterized by unpredictable, transient flares of pain, erythema and swelling in and around the joints [1]. PR is an under-researched condition, particularly with regards to the patient experience. Between flares patients are typically asymptomatic, and laboratory and imaging investigations normal, often leading to diagnostic delays. PR and RA are closely linked, with shared genetic risk factors, a similar distribution of affected joints—the wrists, metacarpophalangeal joints and proximal interphalangeal joints, and a similar prevalence of RA-related autoantibodies; anti-citrullinated protein antibodies (ACPA) and rheumatoid factor (RF) [2, 3]. However, ultrasonography findings indicate inflammation in PR is predominantly extra- rather than intra-articular, suggesting a distinct disease process [4]. Nevertheless, around 50% of patients with PR eventually develop persistent polyarthritis [5, 6], and individuals with PR are considered to be at-risk of developing RA [7].

Evidence from recent clinical trials has demonstrated that therapeutic intervention in at-risk individuals with the aim of preventing RA can reduce the likelihood of RA developing [8], delay onset of RA [9] and reduce the severity of RA if it does develop [10]. At-risk individuals include those who screen positive for ACPA (anti-CCP positive) and RF but without synovitis, those with undifferentiated arthritis, and those with PR [7]. Most people with PR are anti-CCP positive, and this is a risk marker for future progression to RA [11]. As a clinically recognizable pre-rheumatoid state, PR therefore presents a potential window of opportunity for preventative intervention [7]. A systematic review of the efficacy and safety of pharmacological treatments for PR found limited evidence from uncontrolled studies; antimalarial therapy, for example, hydroxychloroquine, may reduce the recurrence of PR flares, help achieve disease remission and could delay development of RA [12], but evidence in support of other conventional/biological disease-modifying anti-rheumatic treatments, and corticosteroids, is minimal. The first randomized clinical trial investigating disease-modifying treatments (abatacept versus hydroxychloroquine) for individuals with PR has shown promising results, preventing both flares of PR and future progression to RA [13]. However, interpreting PR research is likely to remain a challenge due to the lack of consensus on classification or diagnostic criteria for the condition. Importantly, the perspectives of patients living with PR, including their treatment preferences and willingness to engage with different treatments, have not been explored [14], and outcomes measured in PR research may not reflect those that are important and relevant to patients with PR. This could limit the transferability of research findings to clinical practice [15].

This study aimed to explore patients’ perceptions and experiences of PR, including symptoms, impact, treatment outcomes and potential progression to RA.

## Methods

This was a qualitative interview study conducted from a phenomenological standpoint, seeking to understand the lived experience of PR. Ethical approval was obtained from NRES Committee West Midlands—South Birmingham (REC number 10/H1207/98). The study is reported in line with the consolidated criteria for reporting qualitative research (COREQ) checklist (Supplementary Data S1, available at *Rheumatology Advances in Practice* online).

### Participants and recruitment

Participants were recruited from two secondary care rheumatology clinics in the UK (Birmingham, Leeds) from ongoing cohort studies identifying individuals at risk of inflammatory arthritis [16]. Patients were eligible to participate if they were aged 18 or over, able to give informed consent, able to speak and understand English, and had a diagnosis of PR according to a rheumatologist. Patients were approached in-person during their usual outpatient clinics by one of three rheumatologists (K.R., K.M., H.G.) and invited to participate in an interview focusing on understanding their experiences of living with PR.

### Data collection

Data were collected through 1:1 semi-structured interviews. An interview schedule was developed by the research team and two patient research partners (PRPs) (Supplementary Data S2, available at *Rheumatology Advances in Practice* online). The interview schedule was iterative, with new questions added based on participants’ responses in preceding interviews. Interviews were conducted face-to-face in a private room or by telephone, depending on participant preference, by one of two researchers (Z.P.L., MSc, research associate, or R.J.S., PhD and chartered psychologist; both experienced female qualitative researchers with an interest in the perspectives of individuals at risk of developing RA, both unknown to participants) and lasted between 60 and 90 min. Interviews were audio recorded and transcribed verbatim. Reflective notes were made after each interview.

### Data analysis

Data were analysed using reflexive thematic analysis. Transcripts were initially coded by one researcher (L.S.C., female podiatrist and doctoral research fellow with qualitative research experience in the area of RA prevention), who read and reread the transcripts, generated initial codes and collated similar codes to generate preliminary themes. Data were organized in NVivo v.12 (QSR International; 2018). Two transcripts were second coded by another researcher (H.J.S.). Discussions regarding coding decisions and names of themes were held with the wider analysis team (H.J.S., K.M.). Any discrepancies during the analysis process were settled by group consensus.

### Patient and public involvement

Interview questions were piloted with PRPs with PR and revised according to their personal experiences. PRPs were also asked to identify as many initial codes relating to symptoms in the data, by labelling different items they felt were relevant. Codes identified by PRPs were incorporated into the analysis.

## Results

**Figure 1.**
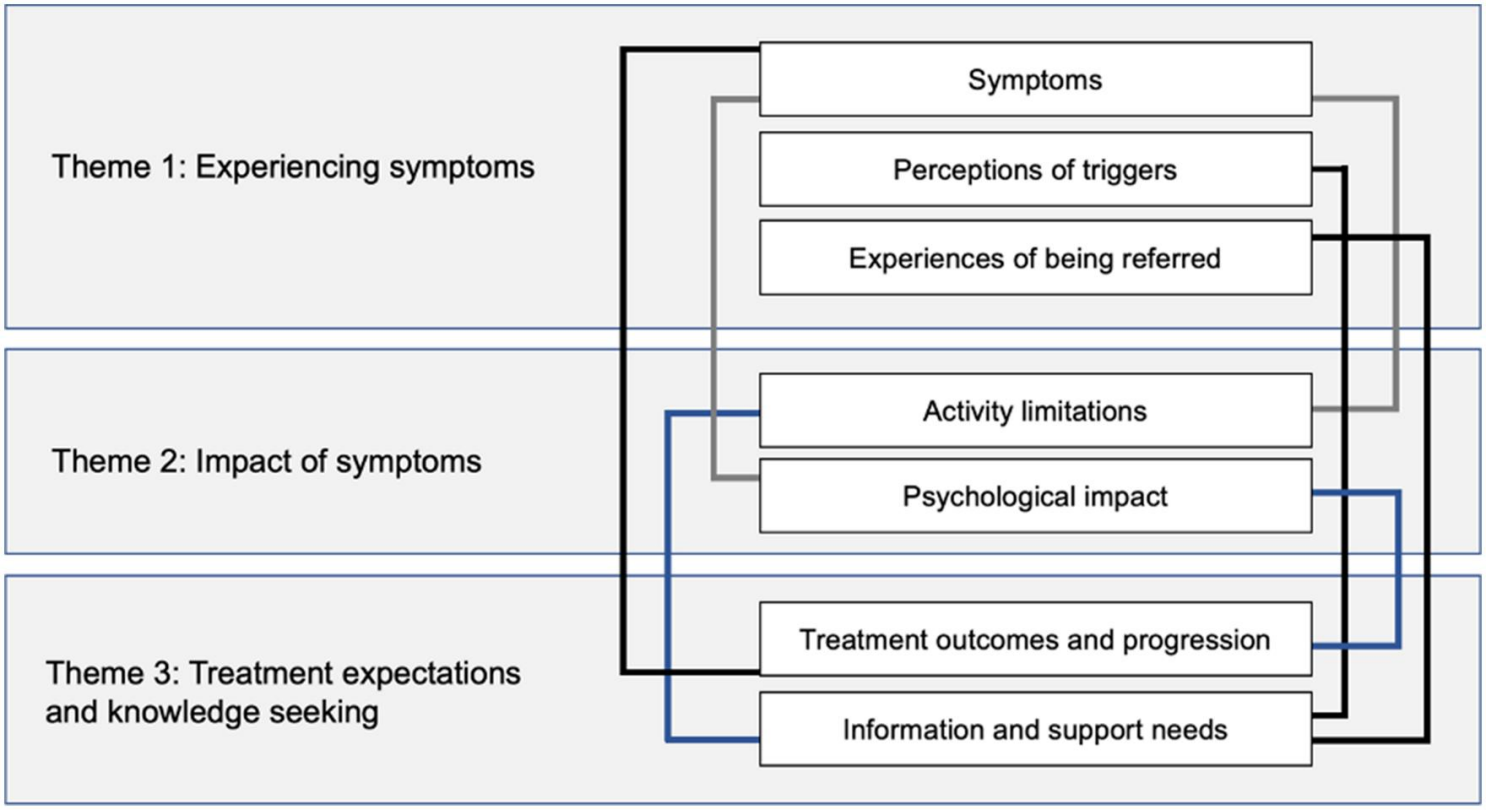
Thematic map

Eight participants (six women) with PR took part in the study. Three participants were anti-CCP positive. PR disease duration ranged from 1 to 30 years. One participant had a first-degree relative with RA. Three themes (seven subthemes) were identified; a thematic map denoting relationships between themes is presented in Fig. 1 and verbatim quotations (Q) supporting each theme are displayed in Tables 1–3.

**Table 1.** Verbatim quotes (theme 1)

Quote no.	Verbatim quote	Participant no.
Q1	‘Intense pain … it’s when you try to move your hands or your elbows it’s as if somebody was trying to force your joints out of place, I dislocated my shoulder many years ago in a fall and it felt very similar to that … my pain threshold is fairly high but when I get these do’s I want to cry I really do because you can’t do anything and number two you can’t even shuffle your bum to get your bum comfy because it’s too painful to move’.	Participant 3
Q2	‘Now, whether it’s an inflammation of the tendon sheathing, or the muscles surrounding it, I don’t know, but I don’t think it’s the joints. I honestly don’t … these feel to be more tendons than bone. They don’t feel like joint. They feel more like tendons in the hands. I think it’s either tendons or muscles, but probably tendons’.	Participant 3
Q3	‘I was falling asleep in the afternoon, I was losing my appetite. I wasn’t doing very much at all, just sitting around really … I’d just fall asleep in the afternoon, but probably for about a couple of h. Well, I’m somebody who’s always rushing around, so it wasn’t me at all. I think I probably lost about eight pounds in weight … I just wasn’t very interested in food. Not like me at all … I wasn’t concentrating as well either’.	Participant 2
Q4	‘It makes you tired … it knocks the stuffing out of you, yeah’.	Participant 4
Q5	‘I think the stress of what I’ve gone through has been a trigger, to be honest. Stress, trauma … I don’t think I do anything that makes it happen or makes it stop if you know what I mean. It just happens’.	Participant 8
Q6	‘They’ve asked me this before, you know, “What brings it on?” and the only two things I can think of is actually stress on that particular joint and tiredness … if I don’t get any sleep then … I tend to have an attack’.	Participant 4
Q7	‘No idea no apart from it seems to happen worse in the winter than it does in the summer. Winter time it seems to be more often and more intense … And it mainly seemed to be either extremely cold weather sort minus 4 or 5 somewhere round there despite the fact that I’m in the warm or a lot of rain I mean we had all those floods I was very bad’.	Participant 7
Q8	‘Perhaps because I’m not an old lady, you know, that it maybe didn’t register that it could be some kind of arthritis. It took a while for me to get that, to that point, to get that referral. And I even, I went back to the GP when I knew the referral was in place, I was having a lot of problems walking—and she, even at that point, one of the GPs at my surgery had said, “Shall we cancel this referral and see how you go?” And I was like that, “No, keep the referral.” So I felt like I had to really push, you know. I had to be there all the time. I had to be really a nuisance before I got the referral. In my mind, I knew it was some kind of arthritis’.	Participant 5
Q9	‘The sooner you find it, the sooner you discover what it is the sooner you can do something about it. Well I’ve been able to do little about it because I’ve not known about it’.	Participant 8
Q10	‘Oh it was quick, I think it was within; well what happened was, I’d not heard anything I think it was about six week and then I had another attack and I went back and then I had an appointment within two weeks’.	Participant 6

**Table 2.** Verbatim quotes (theme 2)

Quote no.	Verbatim quote	Participant no.
Q11	‘Just personal care, you know, I couldn’t … getting dressed, getting undressed, driving the car, you know, just, it massively impacted, sort of, daily life … I couldn’t do anything, sort of, getting dressed and getting in and out of the bath’.	Participant 5
Q12	‘I haven’t got one now [a social life], well obviously it stops me from doing everyday things, so I can’t go, I can’t go out because I can’t physically sort of like, walk and obviously if it’s in the hands, I can’t grip my pint, so I tend you know, I tend not to’.	Participant 12
Q13	‘You can’t plan anything … Never plan anything nowadays. Never plan it because if I plan it, I have to cancel it. We were actually at a point where we actually booked three holidays within a year and we didn’t go on any of them’.	Participant 4
Q14	‘The biggest concern for me is when I can’t pick up my children … if I’ve got pain in a joint in my arms or upper body and I need to pick them up then I will always grit my teeth and do it. I don’t think I’ve ever actually come to a point when I couldn’t physically do it, but it is extremely painful. That’s the bit that bothers me and upsets me the most, because who wouldn’t want to be able to pick up their own child?’	Participant 1
Q15	‘I have had to have quite a bit of time off work, because such as like the job itself involves you know, certain things, and it’s like, I’m right handed so if there’s an attack in my right hand, I can’t even hold a pen, I can’t even lift, so I just can’t typically do the job, you know, do the job’.	Participant 6
Q16	‘I think the way it’s impacted on me the most is … it really depressed me to begin with because … I didn’t have a bloody clue what was wrong, I just knew things didn’t seem to sort of work like they should do anymore, but just sort of suddenly. That got me down a lot. I don’t know. It’s weird, really. I don’t know if it makes you aware of your own mortality or what. It just makes me feel older than I am’.	Participant 8
Q17	‘I mean when I’m not in flare up, then obviously everything is normal and I think that’s the thing that keeps, you know, that keeps me going, but my mood is, when I’m having the flare up, my mood is very, very low to the point that you know, I just sort of like, don’t want to get up or, you know, anything like that. I suppose I feel quite sorry for myself’.	Participant 6
Q18	‘It’s made me a little bit short tempered at times I must admit, I do get very frustrated and very annoyed … I’ve spent h and h and h building models and all sorts of things and had a great deal of patience, I couldn’t do it now because I haven’t go the patience to do it … apart from being annoyed, I’m not being as independent as I would like to be that’s about I can think of saying really’.	Participant 7
Q19	‘I don’t particularly understand it at all and what it means, you know, long time as to what like is going to, you know, going to happen, which scares me … Whether I’m going to have to stop work’.	Participant 6
Q20	‘You’ve got to keep positive about things. We can get all depressed. I don’t know how it’s going to affect me in the future but at the moment I can still do everything really’.	Participant 8

**Table 3.** Verbatim quotes (theme 3)

Quote no.	Verbatim quote	Participant no.
Q21	‘I’m concerned about the medication that I might have to go on. I suppose I’m concerned because that’s possibly going to be my only option, other than trying more diets. I know that some of the drugs are quite harsh and there are lots of side-effects’.	Participant 1
Q22	‘Oh, I hate taking tablets. I really don’t like to, you know. If I can cope, I will, rather than take tablets. I’ve got to be in a bad way… I felt in a really bad way, I think at that time I would have been happy to take the methotrexate. That’s how bad it felt. Yeah, I would have been happy to start the treatment at that point’.	Participant 11
Q23	‘I think with anything, from my point of view, I’d want to know really. The way I look at things, for me anyway, the biggest fear is the fear of the unknown. Once you know what you’re dealing with, whether it’s good or bad in any situation in life, you deal with it because you know what you’re dealing with. I think the fear of the unknown is worse’.	Participant 8
Q24	‘Yes if there was such a thing yes definitely, if they could tell me how it’s going to go, is it going to get worse, is it going to get better, is it going to stay the same, is it going to move to more parts of my body that sort of thing, if there’s tests for that then yes brilliant’.	Participant 7
Q25	‘No, I think it’s better to leave the man in ignorance in these things. If you could tell me …that in five years’ time I am going to have rheumatoid arthritis, and in another five years’ time it will be severely debilitating, then I would say, “Okay. What options are there for me, to either subdue that condition, or remove that condition?” I would be interested in that sort of scenario, but not just to know that I’m gonna get it worse. No, thank you very much. I want to know what you’re going to do… and if you can’t do anything, I wouldn’t want to know’.	Participant 3
Q26	‘I haven’t got a clue [how symptoms will progress] but from what I read on the web about normal arthritis if it’s left untreated it can get worse and literally cripple you up for life. So I’m presuming it’s the same sort of thing’.	Participant 7
Q27	‘Well, I think I’m naturally doing that [receiving support] here, so I don’t need to go and do it again somewhere else really’.	Participant 4
Q28	‘I’d be really keen to understand why it happens rather than just having a medicine to fix it. See if there was a way that you could tackle the underlying reason for it happening rather than masking the symptoms. I’d be really keen to explore whether there was anything I could do to help it, like with diet or exercise or mental state or whatever it might be that could help it … I’ve read a lot about people who do meditation and things like that’.	Participant 1

### Theme 1: experiencing symptoms

#### Symptoms

Symptoms occurring during a PR flare included pain, swelling, stiffness, redness, increased temperature, muscle weakness and fatigue. Pain was typically described as intense and excruciating, likened to that associated with severe physical injury (Q1). Pain and swelling were frequently accompanied by a reddening of the skin around the joint area, described as becoming hot and sore to touch. Swelling occurred around smaller joints rather than larger ones. Participants experienced stiffness in the joint area with a varying degree of intensity, with some participants describing as the joint area locking or seizing up. When severe stiffness occurred, it resulted in a complete loss of function in the affected joint:

*I’m describing it as it’s been in the freezer half an hour, sort of, stiffness, you know. And then in another half hour it’s frozen, and that’s it then, and I’m stuck there, yeah.* (Participant 3)

One participant described the joints as ‘*feeling solid*’, sometimes without pain (Participant 3). In other cases, pain and swelling were primary symptoms while stiffness was a mild experience. Participants also highlighted that tendons, rather than joints, were often the main issue (Q2), and experienced muscle weakness and loss of movement:

*I’ve always been fairly strong in the arm but I can’t grip with my hands nothing like as well as I could … How do I put it, balance wise so to speak, not in a dizziness sense but in the sense that you feel your joints won’t hold you. I remember my left leg afterwards for instance quite often gives way with me.* (Participant 7)

Participants experienced physical and cognitive fatigue during PR flares (Q3, Q4), describing ‘*weariness taking over*’ (Participant 8) and *feeling* ‘*as though you’ve been through an ordeal*’ (Participant 6).

#### Perceptions of triggers

Participants attempted to identify factors that could precipitate symptoms. Some participants felt that they could identify specific triggering factors, including tiredness (Q5), lack of sleep, stress (Q6), diet, alcohol, overexertion, vigorous exercise, placing excessive stress on the joints and infection. Two participants observed an increase in frequency of episodes in the winter (Q7). However, many episodes were believed to be random occurrences with no obvious precipitant:

*At first I thought it was because I was drinking fresh orange juice … so I watched you know, what I ate and what I drank … there’s nothing that you can specifically put your finger on as to what causes it to happen.* (Participant 6)

#### Referral experiences

Participants described delays in being diagnosed with PR, and difficulties in getting a referral to a specialist, attributing this to their age (Q8) or to their symptoms not being severe when presenting to a GP:

*Usually, I go to the doctors as the symptoms start to go off and … ‘it’s a touch of rheumatics, probably the damp weather that’s doing it, here’s some painkillers off you go’ and basically that’s been it.* (Participant 7)

Participants whose referrals had been delayed expressed feelings of frustration and recognized the importance of earlier referral to a specialist (Q9). In contrast, participants who presented to a GP in the middle of a PR flare reported quicker onwards referrals (Q10).

### Theme 2: impact of symptoms

#### Activity limitations

Participants described a reduction in overall function during a flare, primarily due to the intensity of pain. PR symptoms impacted on personal care, for example, getting dressed and going to the toilet (Q11). PR symptoms had a significant impact on walking, recreational and social activities (Q12), and exercise:

*It stops me from exercising on the bad days, which I find frustrating because that’s something I’ve always enjoyed, but obviously if my feet hurt I can’t run and if my arms hurt I can’t do push-ups. There are just certain things that I can’t do.* (Participant 1)

Participants also discussed needing to rely on others during PR flares, cancelling plans and feeling like a burden. One participant recalled taking extra painkillers when away with a group and at a family event ‘*so as not to be nuisance*’ (Participant 2). Participants also described difficulties in planning ahead (Q13), for example, holidays, with subsequent financial impact:

*It has on more than one occasion cropped up and I’ve had to cancel holidays. The last time I had to cancel a holiday it actually cost me £700 … I couldn’t get a penny back, that was it. £700 for me is a terrific amount of money.* (Participant 7)

Participants’ caring responsibilities were also affected, for example, caring for disabled family members and children (Q14). The impact of PR symptoms, particularly pain, fatigue and loss of function, on work were reported (Q15), including difficulty performing job roles, needing time off work and job loss.

#### Psychological impact

Participants described psychological distress as a result of their symptoms; the intense pain and unpredictable, abrupt and intermittent nature of episodes generated feelings of confusion, frustration, low mood and tearfulness (Q16). Most participants described their first episode of PR as particularly shocking, worrisome and confusing because it occurred suddenly and unexpectedly. Most participants worried about the course of their condition and potential outcomes (Q17). Negative emotions were compounded when participants encountered diagnostic uncertainty, could not obtain solutions to help manage their symptoms, and by lack of information:

*Yeah, anti-CCP, and that’s when I went sort of like into panic mode because I didn’t know anything about it, I didn’t know what it was.* (Participant 6)

The impact of PR on participants’ lives also led to psychological distress, causing loss of independence and confidence (Q18). Some participants worried about the future (Q19) and felt nervous about having another flare, feeling ‘*a bit like a time bomb*’ (Participant 5). Other participants were unaffected psychologically and maintained a positive attitude (Q20), with one perceiving that ‘*as you get older, you’re more accepting that things will happen to you*’ (Participant 2), and another feeling ‘*grateful that I’m not in as too bad a state as some people*’ (Participant 1).

### Theme 3: treatment expectations and knowledge seeking

#### Treatment outcomes and progression

Participants discussed taking/being offered pharmacological treatments for PR, including steroids, hydroxychloroquine, sulfasalazine and methotrexate. Participants discussed how some medications (e.g. steroids, painkillers) were masking the pain rather than treating the condition, and that the effects were variable. Some participants had concerns about taking medication for PR, primarily due to side effects (Q21). Perceptions of side effects were influenced by personally knowing or hearing about others on the same drug, and by information on the internet:

*The scary thing for me is, if it would affect my eyes and the liver, kidneys, because they* [other patients on hydroxychloroquine] *do tend to suffer quite a lot from urine infections.* (Participant 6)

However, some participants perceived that worsening symptoms would influence their willingness to take certain drugs, for example, methotrexate (Q22). One participant perceived that he had exhausted all pharmacological options and his only option for the future was steroids.

Most participants conveyed that their ideal treatment outcome was a cure for PR, although they expressed doubt that this was achievable. In the absence of a cure, participants wanted their symptoms to be ‘*under control*’ (Participant 4), to ‘*feel like it’s gone always*’ (Participant 8), and to stop progression. Other important outcomes to participants varied depending on their personal experiences of living with PR, and what symptoms affected them most. These included improved walking, social function and muscle strength.

Most participants wanted to understand how their symptoms would progress (Q23, Q24), and indicated that their willingness to engage with preventive measures depended on to the likelihood of their PR progressing to RA:

*I think with anything, from my point of view, I’d want to know really. The way I look at things, the biggest fear is the fear of the unknown. Once you know what you’re dealing with, whether it’s good or bad in any situation in life, you deal with it because you know what you’re dealing with. I think the fear of the unknown is worse.* (Participant 8)

However, two participants were not interested in learning about progression of their symptoms/condition to RA (Q25), unless they knew there were definitive preventive treatments available:

*No, I think it’s better to leave the man in ignorance in these things … not just to know that I’m gonna get it worse. No, thank you very much. I want to know what you’re going to do… and if you can’t do anything, I wouldn’t want to know.* (Participant 3)

Participants perceived that knowing that the condition would worsen without a solution would ‘*put a bit more doubt in your mind*’ (Participant 4) and ‘*alter your psychological state*’ (Participant 3).

#### Information and support needs

Most participants highlighted a lack of information about PR, particularly in terms of pharmacological treatment options and self-management advice.

*When I saw the consultant a couple of weeks ago, I asked if that* [sulfasalazine] *was an option and he said that it was. I suppose it’s a little bit frustrating that they haven’t suggested that one before. I don’t feel confident that I’ve been told everything that’s available. I’ve had to ask the question and I’ve had to suggest that back to the consultant. It would be good to feel confident that I knew all of my options.* (Participant 1)

In addition to seeking information about medications on the internet, some participants also sought self-management advice and general information about their condition, focusing on RA information in the absence of PR information (Q26). In contrast, two participants refrained from looking at anything on the internet, receiving information and advice solely from medical professionals. In one case, this was because the participant did not own a computer and therefore did not engage with internet searches. In the other case, the participant preferred not to seek information about PR:

*I know nothing about it, other than what they’ve told me here. I don’t look at the internet and find out what the symptoms should be … I mean, I dismiss it as much as I can. I might be foolish for dismissing it and treating it as lightly as I do, because it’s not affecting me, but I prefer to do that and get on with life.* (Participant 3)

Perceptions of PR support groups varied and depended on personal preference (Q27). Participants who had family members with RA discussed seeking information from them. One participant sought support in the form of an emergency number to ‘*ring somebody up and say I’m stuck in a chair, can you come round?*’ (Participant 7). Preferences for the extent of information varied, with some participants wanting to know as much as possible about PR, and others wanting to ignore. Participants who did want information highlighted the desire to understand how they could help themselves (Q28); for example, how they could prevent PR flares or stop progression of the overall condition. To manage and prevent PR symptoms, some participants had trialled lifestyle changes, including cider vinegar and gluten free diets, healthy eating, keeping active, tai chi and joint protection, despite uncertainty around advice. One participant had not made any changes, other than taking medication for PR, due to lack of information about self-management:

*I wouldn’t actually know what to do, to be honest, and nobody’s said anything else*. (Participant 4)

## Discussion

The management of patients with PR is a frequently encountered clinical challenge. This study enhances understanding of the perceptions and experiences of PR among individuals living with the condition. It provides insight into the range and impact of PR symptoms, experiences of referral and diagnosis, perspectives of treatment outcomes, and information and support needs. A range of symptoms experienced by individuals with PR have been identified, some of which (e.g. reduced muscle strength, fatigue and psychological distress) have not previously been identified. These symptoms are also described by those at the earliest stages of RA [17]. However, the fluctuating nature of symptoms in PR, in addition to a lack of understanding about triggers, result in more uncertainty and disruption and subsequent difficulty in planning future events. This study also highlights the severity of PR symptoms, likened by patients to physical trauma or injury, which is often under-appreciated by clinicians and marks PR out as quite different from other pre-RA states [14].

At present, a major difficulty in interpreting PR research is variable inclusion criteria, causing difficulties in comparing clinical, imaging and therapeutic studies, and resulting in a paucity of robust evidence to inform clinical decision making. Consequently, there is a pressing need for consensus-based classification criteria so that future research can be better aligned and more clinically meaningful. The current study has revealed patient-reported PR symptoms that can inform future development of classification criteria for this condition, ensuring the full spectrum of symptoms are considered in a robust consensus process.

People with PR are at risk of progression to RA [7, 18]. Recent evidence from randomized controlled trials (RCTs) in at-risk populations have indicated the potential to delay or prevent progression to RA through pharmacological intervention, but recruitment challenges are common [19–21]. These trials likely included many participants with PR. The feasibility of conducting preventive intervention trials involving individuals with PR similarly depends on the acceptability of preventive interventions among individuals with this condition. Participants in the current study highlighted concerns about taking medication, primarily due to potential pharmacological side effects, but had attempted to, or wanted to understand how to self-manage symptoms, for example, through lifestyle changes such as diet or activity. These findings reflect previous qualitative studies, where healthy eating, increased exercise, smoking cessation and oral health maintenance with the aim of preventing RA were perceived to be more acceptable than preventive medication [14, 22]. Also, in congruence with these previous studies, current findings suggest that medication is potentially more acceptable to people with PR when symptoms are more severe, and willingness to engage with preventive measures was influenced by the likelihood of PR progressing to RA.

There is a clear need for future robust trials to determine the effectiveness for treatments for PR. To maximize transferability of PR research findings to clinical practice, outcomes in future PR clinical trials should be measured consistently and reflect what is important to patients [15]. In addition to symptom management and stopping progression, this study highlights the impact of PR on activity limitations and mental health; these outcomes should not be overlooked by researchers or clinicians.

There is increasing interest in prediction of RA. In previous studies, some individuals at risk of developing RA perceived predictive testing as useful to clarify their risk status, and to prepare mentally and physically for the future, while others were fearful and anxious about predictive testing, suggesting it would reduce their ability to enjoy life [13, 22–25]. In congruence, the current study indicated that some participants wanted to understand how their PR symptoms would progress, whereas a minority of participants did not want to know, particularly if there were no effective treatments. A previous qualitative ideal-type analysis indicated that an at-risk individual’s personality and overall orientation to health behaviours impacted on their attitude towards preventing RA; for example, fully engaging with preventive measures versus not wanting to worry about the future and dealing with things as they happen. Findings from the current study corroborate that individuals with PR might need differing levels of information and support relating to PR and prevention of RA.

Findings from this study must be viewed in light of several limitations. First, symptoms were reported retrospectively and, in some cases, there was a long interval between symptom onset and the interview. This was inevitable, as some participants experienced a delay in diagnosis after seeking help for symptoms of PR, reflecting the lack of accepted diagnostic/classification criteria for PR. Notwithstanding, the use of a broad definition of PR to select participants ensured the range of symptoms that constitute PR were explored. Second, while small sample sizes are common in qualitative research, interviewing further participants may have provided additional insights. Nevertheless, this is the first study to explore experiences of PR from the perspective of individuals living with the condition and provides a grounding for further qualitative research in this area, particularly in the context of being at risk of RA and potential prevention. The interviews conducted provided detailed information that directly addressed the aim of the study and allowed participants’ journeys to be explored in depth. Finally, individuals with PR who were unable to speak and understand English were excluded from this study to ensure that participants could participate fully in the interviews. Future research could explore experiences of PR among a more diverse study population.

## Conclusion

This study has provided early insights into what is important for patients with PR, reflecting the severe and unpredictable course of their symptoms and difficulties and delays in obtaining a diagnosis. PR has a significant impact on patients and recognizing the need to enable patients to access healthcare in flares, particularly during the initial presentations, may prompt earlier referral to rheumatology specialist services. Further work to standardize classification criteria and outcome measurement in PR is critical to facilitate meaningful clinical trials in this area.

## Supplementary Material

rkaf138_Supplementary_Data

## Data Availability

The data that support the findings of this study are not publicly available as they contain information that could compromise the privacy of research participants.
