# Supplementary material for: Living with palindromic rheumatism: a qualitative interview study
Source: Rheumatol Adv Pract. 2025 Dec 2;10(1):rkaf138. doi: 10.1093/rap/rkaf138 (PMC12758112; doi:10.1093/rap/rkaf138)
Supplement: rkaf138_Supplementary_Data [file rkaf138_supplementary_data.zip › PR_supplementary_file_2_151025.docx]

**Supplementary Data S2. Interview schedule**

1. Tell me about how you symptoms started..... What were your initial symptoms of palindromic arthritis? (fatigue, joint pain, morning stiffness and joint swelling). What symptoms were worst for you? What symptoms drove you to seek help?
2. What has been the impact of your symptoms?
3. What are you views about your diagnosis?
4. Tell me what you know about palindromic arthritis?

PROMPTS: What do you think the causes of your symptoms could be? What do you think the risks factors for condition are? Tell me about how serious you think your illness is? What would be the impact of your symptoms been on your life? Do you think you would be able to control your symptoms yourself? Do you think there are treatments available that would effectively control your symptoms? What did you think may have caused the symptoms? How did this influence the way that you and others responded?

1. How did you and others react to your initial symptoms of RA?
2. How long did you wait before seeking help?
3. What was your experience of seeking help from the GP?
4. What is your relationship like with your GP? Is he or she approachable?
5. Did you worry about what the doctor might say, or how he or she may have treated you when you went to seek help?
6. Do you feel your GP referred you to a specialist in enough time? (or did you experience a delay)
7. How stressful have you found your experience of accessing care so far? Do you think it has had an impact on your mental or physical health?
8. What are your views about your treatment?
9. What are your views about the services available to people with palindromic arthritis?
10. Do you have enough information about your condition?
11. How do you think your symptoms will progress?
12. What are your concerns or worries about your illness?
13. I appreciate the time you took for this interview. Is there anything else you think would be helpful for me to know?
